# Nanoemulsions Based on Sunflower and Rosehip Oils: The Impact of Natural and Synthetic Stabilizers on Skin Penetration and an Ex Vivo Wound Healing Model

**DOI:** 10.3390/pharmaceutics15030999

**Published:** 2023-03-20

**Authors:** Cynthia Nara Pereira Oliveira, Marcel Nani Leite, Natália Aparecida de Paula, Yugo Araújo Martins, Sonia Aparecida Figueiredo, Marco Andrey Cipriani Frade, Renata Fonseca Vianna Lopez

**Affiliations:** 1 School of Pharmaceutical Sciences of Ribeirão Preto, University of São Paulo, Ribeirão Preto 14040-903, SP, Brazil; 2 Dermatology Division of Department of Internal Medicine, Faculty of Medicine of Ribeirão Preto, University of São Paulo, Ribeirão Preto 14049-900, SP, Brazil; 3 Department of Food and Drug, School of Pharmaceutical Sciences, Federal University of Alfenas, Alfenas 37130-001, MG, Brazil

**Keywords:** lipid nanoemulsion, wound healing, vegetable oil, human skin ex vivo model

## Abstract

Vegetable oils offer excellent biological properties, but their high lipophilicity limits their bioavailability. This work aimed to develop nanoemulsions based on sunflower and rosehip oils and to evaluate their wound-healing activity. The influence of phospholipids of plant origin on nanoemulsions’ characteristics was investigated. A nanoemulsion prepared with a mixture of phospholipids and synthetic emulsifiers (Nano-1) was compared with another prepared only with phospholipids (Nano-2). The healing activity was evaluated in wounds induced in human organotypic skin explant culture (hOSEC) based on histological and immunohistochemical analysis. The hOSEC wound model was validated, showing that high nanoparticle concentration in the wound bed interferes with cell mobility and the ability to respond to the treatment. Nanoemulsions were 130 to 370 nm, with a concentration of 10^13^ particles/mL, and a low potential to induce inflammatory processes. Nano-2 was three times larger than Nano-1 but less cytotoxic and could target the oils to the epidermis. Nano-1 permeated intact skin to the dermis and showed a more prominent healing effect than Nano-2 in the hOSEC wound model. Changes in the lipid nanoemulsion stabilizers impacted the cutaneous and cellular penetration of the oils, cytotoxicity, and healing kinetics, resulting in versatile delivery systems.

## 1. Introduction

Vegetable oils have excellent biological properties, such as antioxidant, anti-inflammatory, and antimicrobial action. These properties are potentially interesting for treating skin diseases requiring pro-healing stimuli, such as burns and ulcers. The oils’ properties vary depending on the diversity of active components found in each plant species, which usually act synergistically, increasing their therapeutic potential [1]. Two vegetable oils with exciting characteristics for wound healing are those extracted from sunflowers and rosehips.

Sunflower oil is a lipid mixture extracted from the seed of the species of *Helianthus* [2]. It contains antioxidant [3], antimicrobial [2], and anti-inflammatory [4] substances. The sunflower oil’s main lipid component is linoleic acid in the form of triglycerides (61.5%), but it also contains oleic acid (24.3%), palmitic acid (9.3%), stearic acid (3.7%), and linolenic acid (1%) [4]. Distilled sunflower oil also contains 5% phytosterols and 1% vitamin E, substances with high antioxidant activity [5]. The similarity of the lipid content of sunflower oil with the cutaneous lipid matrix stimulates the production of epidermal ceramides, cholesterol synthesis, and activation of peroxisome-proliferator-activated receptors alpha-type (PPARa), which play an essential role in cell differentiation regulation and are involved in skin barrier homeostasis and inflammatory process [5].

Rosehip oil is another oil extracted from plant seeds of the *Rosacea* family with pronounced antioxidant, anti-inflammatory, and replenishing activities with respect to skin lipid constituents [6]. Its composition is rich in phenols (flavonoid glycosides and proanthocyanidins), vitamins E and C, and carotenoids, contributing to its antioxidant and protective action on cells in the process of re-epithelialization [7]. Its fatty constituents vary according to the species. They are composed of between 43 and 49% of linoleic acid, 32 and 38% of linolenic acid, 14 and 16% of oleic acid, 3 and 5% of palmitic acid, 0.1 and 5% of palmitoleic acid, and 1 and 2% of stearic acid, in addition to other fatty acids present in concentrations lower than 1%, such as lauric acid, myristic acid, arachidonic acid, gadoleic acid, and behenic acid [8]. These fatty acids can increase cell membrane permeability and facilitate the entry of growth factors, stimulating cells’ proliferation, migration, and neoangiogenesis, thus acting directly in the proliferative wound-healing phase [9].

Sunflower and rosehip oils, therefore, have essential properties to stimulate and modulate the healing process of skin wounds [10]. However, the high lipophilicity of oils negatively impacts their administration and bioavailability. Pharmaceutical forms that allow the administration of these oils’ mixture and good compatibility with the biological environment should contribute to expressing the desired oils’ activities synergistically and concomitantly. In this context, nanoemulsions emerge as an exciting delivery system for vegetable oils. In addition to increasing the apparent solubility of lipophilic active compounds, nanoemulsions have good thermodynamic stability, ease of manufacture, and excellent kinetic stability [11]. They can also sustain the release of active substances, preventing their immediate degradation in the wound bed and thus increasing their bioavailability. Consequently, it is possible to reduce the administered dose, adverse effects, and treatment cost [12].

Nanoemulsions can be defined as heterogeneous metastable drug-delivery systems in which a liquid is dispersed in the form of nanometric droplets in the internal phase of another liquid, stabilized by an emulsifying system [11], whose formation is dependent, in addition to the composition and proportions, on the method of preparation [13]. Nanoemulsions’ emulsifiers and stabilizers can interfere with the final characteristics of the nanoemulsion and, as such, may change the way oils and their components are delivered to the skin. Therefore, we hypothesize that these modifications in the nanoemulsion’s characteristics caused by stabilizers can impact the oils’ healing efficiency.

Phospholipids can be used as nanoemulsions’ emulsifiers and stabilizers and as replenishers of the skin’s lipid content. In addition to their structural function, phospholipids can intervene in several metabolic processes, such as the active process of phosphorylation, mitosis, cellular organization, ionic exchanges, and the synthesis of biologically active compounds, such as prostaglandins and leukotrienes from arachidonic acid [14]. Moreover, they can be extracted from natural sources, such as the sunflower itself, which, as previously described, has several potentialities as a healing agent.

Both the composition and dimensions of nanoemulsions influence the bioavailability of the substances that are part of it [15] and, consequently, the therapeutic response. Thus, this work focused on the impact of changes in nanoemulsions’ emulsifying system on vegetable oils’ skin penetration and healing capacity. Nanoemulsions based on sunflower and rosehip oils as an internal phase were stabilized with phospholipids derived from plants with or without added synthetic surfactant. This way, nanoemulsions with different stabilizers were obtained using the same vegetable oils as the internal phase. Their potential for cutaneous administration was evaluated as a function of the nanoemulsions’ characteristics and composition.

Furthermore, the wound-healing potential of nanoemulsions was investigated for the first time using an ex vivo wound model induced in human organotypic skin explant in culture (hOSEC). The influence of the nanoemulsion’s number of particles on keratinocyte viability, proliferation, and migration was verified. The particle number suitable for carrying out the wound healing experiments was standardized.

The sunflower and rosehip oils’ nanoemulsion formulations stabilized only with natural-origin phospholipids were optimized using a Quality-by-Design approach, and the influence of the low concentrations of a non-ionic synthetic surfactant on the physicochemical and biological properties of nanoemulsions was carefully evaluated. The appropriate combination of phospholipids of natural origin allowed for obtaining versatile nanoemulsions of vegetable oils, favoring the treatment of of cutenous wounds and other deep-layers seated skin diseases.

## 2. Materials and Methods

### 2.1. Materials

Sunflower seed oil, rosehip oil, and phospholipids extracted from sunflower (H), containing phosphatidylcholine at 20% (Lipoid H20) and 100% (Lipoid H100), and those extracted from soybean (S), containing 75% phosphatidylcholine (Lipoid S75), were kindly provided by Lipid Ingredients & Technology (Ribeirão Preto, SP, Brazil). Alpha-bisabolol was provided by Atina (Pouso Alegre, MG, Brazil). Lipophilic 4,4-Difluoro-1,3,5,7-Tetramethyl-4-Bora-3a,4a-Diaza-s-Indacene (BODIPY) was synthesized and gifted by collaborators. Polysorbate 80 (Tween 80), polysorbate 60 (Tween 60), sorbitan monooleate (Span 80), glycerin, polyvinyl butyral (Pioloform), propidium iodide, 3-[4,5-dimethylthiazol-2-yl]-2,5-diphenyl tetrazolium bromide (MTT), IL-6 and TNF-α, Histopaque^®^-1077, 3,3-diaminobenzidine tetrahydrochloride, E. coli liposaccharide (LPS), bovine serum albumin (BSA), and Entellan™ Mounting Medium were purchased from Sigma-Aldrich (St. Louis, MO, USA); DAPI (4′,6′-diamino-2-phenyl-indole), fetal bovine serum (FBS), and RPMI-1640 medium from Thermo Fisher Scientific (Hampton, NH, USA); dimethylsulfoxide (DMSO) from Labsynth (Diadema, SP, Brazil); Dulbecco’s Modified Eagle Medium (DMEM) from Gibco-Thermofischer (Waltham, MA, USA); Multi-Analyte Elisa Array Kit from Qiagen (Hilden, Germany); cytokeratin 10 (Ck10); Ki-67 protein from Santa Cruz Biotechnology (Santa Cruz, CA, USA); and NovoLink Polymer Detection System Kit from Novocastra Laboratories Ltd. (Newcastle upon Tyne, UK).

### 2.2. Development of Nanoemulsions Based on Sunflower and Rosehip Oils

Nanoemulsions were prepared with 15% sunflower and 3% rosehip oils, in addition to 0.3% of the antioxidant alpha-bisabolol as the oil phase. The aqueous phase comprised 5% glycerin and enough water for 200 g. As emulsifying agents, up to 15% of synthetic emulsifiers and phospholipids extracted from vegetables (Nano-1) or a combination of phospholipids extracted from vegetables (Nano-2) were used. Nano-1 was prepared with polysorbate 60 (3%) as a synthetic emulsifier and with phosphatidylcholine extracted from sunflower (H100) (0.5%) and soybean (S75) (1.0%). As for the Nano-2 emulsifier system, the synthetic surfactant was replaced by Lipoid H20 (H20), which, like H100, is extracted from sunflower but with a lower phosphatidylcholine content and lower cost.

The proportion between the components of the Nano-2 emulsifying system was defined based on a Box–Behnken-type experimental design [16] with 3 factors at 3 levels, based on 15 randomized experiments, as described in Table 1. The influence of different combinations of phospholipids (independent variables) was analyzed considering the following dependent variables (or responses)–particle size, polydispersity index (PDI), emulsified percentage, viscosity, pH, and cost–according to the methods described in item 2.3. Analysis of variance (ANOVA) was performed to identify the main effects and interactions between variables. Those responses with a significance level (*P*) lower than 0.05 were considered statistically significant. The most appropriate mathematical model was obtained based on the *P* values, predicted and adjusted R^2^. The software used for the analysis was the Design-Expert 11.1.0 © 2018 Stat-Ease.

Using the mathematical model and equations obtained from the experimental design, numerical optimization was performed using the software desirability function to define the percentages of each emulsifier necessary to obtain the desired nanoemulsion. Restrictions were established on the variables that significantly impacted the responses. Therefore, the need to obtain fluid nanoemulsions with viscosity in the range of 61 to 253 cP with minimal cost was established. The optimal concentrations predicted by the mathematical model were then used to experimentally prepare the desired optimized formulation in triplicate and validate the model based on the characterization of the optimized nanoemulsion and comparison with the predicted data.

Nanoemulsions were prepared at 75 °C, dispersing emulsifying agents in the aqueous phase under stirring at 15,000 r.p.m for 5 min, using a high-shear homogenizer (Ultra Turrax, IKA, Guangzhou, China). The oil phase, also at 75 °C, was poured under the aqueous phase. The formed pre-emulsion was submitted to a high-pressure homogenizer (GEA Niro Soavi, Panda 2K NS 1001L, Düsseldorf, Germany) with 800 bar and 3 homogenization cycles.

A control nanoemulsion (NC), without vegetable oils and containing only synthetic surfactants, composed of 18% mineral oil as the oil phase and a mixture of polysorbate 80 (2.5%) and Span 80 (2.5%) as synthetic emulsifiers, was prepared in the same way as the other formulations and used as a control in cell culture and ex vivo wound-healing studies. The concentration of mineral oil used was the sum of the other oils used to maintain the percentage of the oil phase in the formulation. It was developed only as an experimental control of a nanoemulsion prepared with components devoid of biological properties in wound healing.

### 2.3. Characterization and Stability of Lipid Nanoemulsions

Nano-1 and optimized Nano-2 (Table 2) were evaluated for their organoleptic aspects, droplet size, pH, morphology, and variations in these parameters under thermal and physical stress to investigate the stability of each system.

#### 2.3.1. Freeze/Defrost Cycle

The nanoemulsions were kept at 45 ± 2 °C and −5 ± 2 °C for 12 days in 6 cycles of 24 h at each temperature, and then the following characteristics were analyzed: appearance, color, odor, pH, and phases separation.

#### 2.3.2. Centrifugation

Each emulsion was weighed in graduated flasks and subjected to centrifugation (Thermo Scientific, Osterode, Germany) at 3000 r.p.m for 30 min at room temperature and visually evaluated for phase separation.

#### 2.3.3. Emulsification Rate

Twenty-four hours after the preparation of the emulsions, they were centrifuged in graduated flasks as described in Section 2.3.2. The emulsification rate, also known as the creaming index (CI), was calculated using the following equation: CI = (H_S_/H_E_) × 100, where H_E_ is the total height occupied by the emulsion in the graduated flask, and H_S_ is the height of the serum layer after centrifugation [17].

#### 2.3.4. Droplet Size, Polydispersion Index (PDI), and Particle Number Analysis

Size and PDI were determined by photon autocorrelation spectroscopy (DLS) using the Zetasizer Nano ZS 90 (Malvern Instruments, Malvern, UK) at 25 °C. Nano-1 and Nano-2 were diluted 100 and 2000 times, respectively, to ensure sample transparency. Particle concentration was determined using the NanoSight NS 300 nanoparticle tracker (Malvern Instruments, Malvern, UK) and the “NTA 3.1 Nanoparticle Tracking Analysis” software. Formulations were diluted 50,000 times for this analysis.

#### 2.3.5. pH

A digital pH meter (Digimed, São Paulo, Brazil) was used to analyze all preparations manipulated in the present work.

#### 2.3.6. Determination of Apparent Viscosity

Apparent viscosity was determined using the LBDV-III Ultra axial cylinder rheometer—(Brookfield Engineering Laboratories, Middleborough, MA, USA) coupled to the SC4-25 spindle for formulations F2, F4, F6, F8, F9, and F12 described in Table 1 and coupled to the SC4-18 spindle for other formulations. The temperature was 25 ± 2 °C. The shear rates for analyses with spindle SC4-25 and SC4-18 were, respectively, 11 s^−1^ and 12 s^−1^.

#### 2.3.7. Morphology

Samples of nanoemulsions were deposited on nickel metal grids (200 mesh) coated with Pioloform and added with 2% uranyl acetate as negative contrast. After 24 h of drying at a temperature of 25 °C, the samples were observed in a transmission electron microscope (TEM) (JEOL 100CX II, Akishima, Tokyo, Japan) with a voltage of 80 kV and magnification of 20, 50, and 100 thousand times.

### 2.4. Viability and Cellular Uptake

Both assays were performed using representative skin cell lines, NIH-3T3 fibroblasts, and human keratinocytes (HaCaT). The strains were maintained under cryopreservation in liquid nitrogen, in 5% DMSO and 95% inactivated SBF. The cells were defrosted and expanded in cell culture flasks to carry out the experiments using a complete DMEM medium. The cells were kept in an incubator at 37 °C in an atmosphere of 95% O_2_ and 5% CO_2_.

#### 2.4.1. Cytotoxicity

The viability of fibroblasts and keratinocytes in contact with nanoemulsions was evaluated with the MTT colorimetric method [18,19]. In general, cells were plated in 96-well plates at 4.0 × 10^3^ cells/well in a DMEM culture medium, incubated for 24 h at 37 °C in an incubator with 5% CO_2_. After this period, the same volume of 200 μL, but with different concentrations of Nano-1, Nano-2, and NC, ranging from 1.0 × 10^12^ to 2.5 × 10^11^ particles/mL, was added to the culture medium and incubated for 24 h and 48 h before MTT analysis. Controls were performed without formulations to verify any interference in the colorimetric analysis.

#### 2.4.2. Evaluation of Cell Uptake as a Function of Incubation Time

Flow cytometry and confocal laser scanning microscopy evaluated the cellular uptake of nanoemulsions by fibroblasts and keratinocytes. The fluorescent lipophilic dye BODIPY [20] was solubilized in the oil phase of the nanoemulsions at 1 mg/mL of the final formulation.

For analysis by flow cytometry, cells were plated in 24-well plates at a density of 8 × 10^4^ cells/well and incubated for 24 h at 37 °C in an atmosphere containing 5% CO_2_. After this period, cells were washed with saline solution and treated for 5 or 15 min with the nanoemulsions diluted 100 times in the DMEM culture medium, i.e., at 6.5 × 10^11^ particles/mL. Note that the nanoemulsions were not cytotoxic to cells at the particle concentration and time evaluated in this study. After treatment, cells were washed five times with saline solution and trypsinized with 200 μL of 0.25% trypsin solution. Then, the trypsin action was neutralized by adding 200 μL of culture medium, and the cells were collected in conical tubes. For analysis in the flow cytometer (BD FACSCanto II, BD Biosciences, San Jose, CA, USA), 2 μL of propidium iodide (PI) was added at 50 μg/mL as a marker of dead cells. The PI labeling was visualized at λ_exc_ = 488 nm and λ_em_ = 630 ± 22 nm, and the BODIPY labeling at λ_exc_ = 470 nm and λ_em_ = 512 ± 20 nm.

For cell uptake analysis by confocal microscopy, sterilized circular slides 20 mm in diameter were placed on the bottom of each well of 24-well plates. The cells were plated on the slides at a density of 8 × 10^4^ cells/well and incubated for 24 h at 37 °C in an atmosphere containing 5% CO_2_. After this period, the cells were treated for 15 min with the nanoemulsions diluted 50 times, i.e., at 1.3 × 10^12^ particles/mL, in the culture medium. It should be noted that just 15 min of treatment of cells with this concentration of nanoparticles did not cause cytotoxicity. Then, the supernatant was removed, and the cells were washed three times with saline solution, followed by the addition of 500 μL of 1% paraformaldehyde solution, for 15 min, in each well for fixation. After fixation, the paraformaldehyde was removed, followed by successive washes with saline solution. The coverslips, containing the fixed and washed cells, were removed from the wells and placed on a histological slide containing a drop of Prolong Diamante mounting medium with DAPI as a nucleus marker. The slides were protected from light for 24 h before visualization under a confocal laser scanning microscope (Leica, TCS SP8, Wetzlar, Germany). Images were obtained at 63× magnification using an oil immersion objective. For visualization of the nucleus (DAPI), the laser was used at λ_exc_ = 405 nm and λ_em_ = 409 to 514 nm, and for visualization of the formulations (BODIPY), it was used at λ_exc_ = 470 nm and λ_em_ = 512 nm.

### 2.5. Mediators’ Expression of the Inflammatory Process

The influence of Nano-1 and Nano-2 on the expression of inflammatory mediators IL-6 and TNF-α was investigated in human macrophages extracted from 3 different participants (project approved by the Research Ethics Committee (CEP) of FCFRP-USP, protocol CEP/FCFRP No. 506—CAAE: 11726919.0.0000.5403, CEP report No. 3.712.502). Peripheral venous blood (20 mL) was collected in heparinized tubes and added to 20 mL of 0.9% saline solution. Fifteen milliliters of the resulting suspension was transferred to tubes containing 3 mL of Histopaque-1077 solution and centrifuged at 400 g for 30 min at 25 °C. After centrifugation, the mononuclear cells were carefully collected and washed twice in 0.9% saline solution and centrifuged at 400× *g* for 30 min at 25 °C. The pellet obtained was resuspended in 2 mL of RPMI-1640 medium and counted in a Neubauer chamber after Turks staining. The cell suspension was then diluted to 2 × 10^6^ cells/mL density in RPMI medium containing 2.5% FBS. A 500 μL of the suspension was transferred to 24-well plates and incubated for 2 h at 37 °C and 5% CO_2_. After this period, the adhered cells were recognized as macrophages, and the monolayer culture was used for experimentation.

The macrophage culture was treated with nanoemulsions at a concentration of approximately 3 × 10^11^ particles/mL, diluted in the culture medium. As a positive control, the inflammatory state of macrophages was induced by incubating the cells with 10 ng/mL of bacterial LPS [21]. After the treatments, the cells were incubated for 24 h at 37 °C and 5% CO_2_. IL-6 and TNF-α production was evaluated by ELISA, using the Multi-Analyte Elisa Array Kit.

### 2.6. In Vitro Penetration Studies Using Human Skin

Franz-type vertical diffusion cells were used for the penetration test. The volume of the receptor solution was 16 mL. The penetration area was 0.95 cm^2^, and 1 mL of each tested formulation was used. The experiments were performed at room temperature (25 °C). Human skin fragments from abdominoplasty were used as the membrane (CEP/FCFRP protocol nº. 506—CAAE: 11726919.0.0000.5403, CEP report nº 3,712,502). The fragments of subcutaneous tissue were discarded to preserve the dermis and epidermis, and the skin fragments were stored at −20 °C until use. Immediately before the penetration experiments, the fragments were removed from the freezer and defrosted. The vertical diffusion cell was built with the skin positioned between the donor and recipient compartments, with the stratum corneum facing the donor. The donor was filled with 1 mL of Nano-1 and Nano-2. Preparations were labeled with 1 mg/mL of BODIPY. In contact with the dermis, the receptor compartment was filled with isotonic phosphate-saline buffer at 10 mmol/L pH 7.4 (PBS). After 1 h of contact with the skin, the formulation was removed, and its excess was washed off in PBS. Then, the skin fragments were frozen at −80 °C in Tissue Tek, cut to a thickness of 20 µm in a Cryostat (Leica CM1860, Germany), placed on glass slides and coverslips, fixed with Fluoromount, and analyzed in confocal laser scanning microscope (Leica SP8, Germany) at λ_exc_ = 470 nm and λ_em_ = 512 nm with a 20× objective.

### 2.7. Ex Vivo Studies in Human Organotypic Skin Explant Wound Model (hOSEC)

The skin was obtained with the informed consent of patients undergoing plastic surgery (CEP/FCFRP nº. 506—CAAE: 11726919.0.0000.5403, CEP report nº 3.712.502). The inclusion criteria for the study participants who donated skin fragments were women aged 25 to 47 years, non-smokers, non-drinkers, who do not have comorbidities, and who were undergoing abdominoplasty. After removing excess subcutaneous adipose tissue, the explants were cut into 1 cm^2^. In each fragment, a circular excisional ulcer with a diameter of 4 mm was created using a surgical scalpel (punch) currently used for a skin biopsy. Ulcerated skin fragments were placed on filter paper supported by metal grids. The dermis was kept in contact with the filter paper, while the stratum corneum was positioned upwards. In this arrangement, the fragments were cultured in standard 6-well plates containing 5 mL of complete DMEM (supplemented with 10% FBS, 1% solution containing 10,000 units penicillin, 10 mg of streptomycin, 25 μg of amphotericin B, and 1% L-glutamine) at 37 °C in 5% CO_2_ humidified air. The epidermis, therefore, was above the medium/air interface [19,22].

The first series of experiments were performed with different concentrations of Nano-1 to validate the hOSEC wound model and evaluate the influence of the number of particles on the speed of wound closure and cell viability. The explants were treated daily, for up to 14 days, with 5 μL of concentrated Nano-1 and diluted 50, 100, and 200 times, resulting in formulations with 6.5 × 10^13^ to 3.2 × 10^11^ particles/mL.

A set of experiments were then performed to evaluate the formulations’ influence on the kinetics of ulcer closure by treating the skin fragments with the Nano-1, Nano-2, and NC formulations at a concentration of 3.2 × 10^11^ particles/mL daily for 7 days. The culture medium was changed every 3 days, and the tests were performed in triplicate. After the treatment, the explants were collected, fixed in formalin, embedded in paraffin, and stained for histological and immunohistochemical analysis [23].

#### 2.7.1. Histological Evaluation

For histomorphological analysis, paraffin sections (3 μm) were stained with hematoxylin and eosin (HE). The morphology of the epidermis was analyzed qualitatively using the optical microscope LEICA^®^ DM-4000B with the camera LEICA^®^ DFC 280 connected to the computer with the software LAS^®^—Leica Application to capture images (Leica Microsystems, Wetzlar, Germany) [24]. The wound closure was quantitated using the difference between the ulcer diameter in the slice image subtracted from the sum of the extent of reepithelialization at the edges of the lesion (keratinocyte tongue), using ImageJ 1.51 m9 software (National Institutes of Health, Bethesda, MD, USA).

#### 2.7.2. Immunohistochemical Evaluation

Three-micrometer paraffin sections were submitted to antigen retrieval by autoclaving in citrate buffer, pH 6, for 5 min. Endogenous peroxidase was blocked with 3% hydrogen peroxide in PBS, followed by nonspecific blocking with 1% BSA. Sections were incubated with the primary antibody in 1% BSA overnight at 4 °C. The antibodies used were Ck10 (1:100) and Ki-67 (1:100), detected with the NovoLink polymer detection kit and stained with tetrahydrochloride of 3,3 diaminobenzidine. Sections were counterstained with Mayer’s Hematoxylin and mounted in Entellan Rapid Microscopy Mounting Medium. Ck10 and Ki-67 expressions were analyzed qualitatively.

### 2.8. Statistical Analysis

The obtained results were subjected to appropriate statistical analysis, including Response Surface Regression (Design-Expert 11.1.0 © 2018 StatEase), t-test, and One-Way ANOVA, followed by Tukey’s test, using GraphPad Prism 5.0 (GraphPad Software, Boston, MA, USA). Specific tests are described in the items that concern them.

## 3. Results

### 3.1. Nanoemulsion Development

#### 3.1.1. Experimental Design for Preparing Nano-2 Formulation

Table 3 presents the physicochemical characteristics of the formulations obtained with different combinations of phospholipids extracted from a natural source (H20, H100, and S75) used to emulsify sunflower and rosehip oils, as described in Table 1.

The nanoemulsions presented droplets with average sizes ranging between 304 and 403 nm. The droplet size distribution (PDI) ranged from 0.197 to 0.430. However, the majority had a narrow distribution, less than 0.3. The pH of the nanoemulsions ranged from 4.3 to 6.1. The apparent viscosity varied considerably, from 61 cP to 5819 cP. These differences resulted in using two different spindles to determine viscosity. The formulations emulsifying systems cost an average of USD 254.52 ± 30.65 per Kg.

To understand the influence of the selected variables on the characteristics of the nanoemulsions, the values obtained from the dependent variables were submitted to statistical analysis of variance (ANOVA) and mathematical modeling. The statistical significance of each dependent variable is presented in the supplementary material (Table S1), as well as the model that best describes the contribution strength of each independent factor in the dependent responses evaluated. The predictive models found for the dependent variables emulsification rate, mean size, PDI, and pH were not significant (*P* > 0.05), indicating that, for these variables, the general mean is a better response predictor than the mathematical model. For the dependent variables that showed statistical significance, viscosity, and cost, the mathematical model that showed good values of adjustment statistics was the linear one, with Log transformation in base 10 for viscosity. Table 4 presents the results of the ANOVA analysis for these models, terms of the equation and lack of fit (F value), and a summary of the model equations and the adjusted and predicted correlation coefficients.

The values of F and *P* prove that they are significant for viscosity and cost. The predicted and adjusted R^2^ values show a difference between them of less than 0.2, suggesting the adequacy of the values and the absence of block effects (Software Design-Expert 11.1.0 © 2018 Stat-Ease). Three-dimensional and response surface plots illustrating the effect of independent variables on viscosity are provided in the supplemental material (Figure S1).

The formula used to prepare Nano-2 was optimized based on the equations obtained in the experimental design and the desired characteristics. Thus, a viscosity range that could allow the delivery of the formulation through a spray device was defined. Values above 253 cP were not experimentally satisfactory for packaging the formulations in a spray device. Therefore, the defined viscosity range was 61 to 253 cP. The cost was minimized. Using mathematical equations and numerical ranges for viscosity and cost minimization, the desirability value obtained was found to be 0.921. The predicted optimal concentrations to obtain the optimized nanoemulsion were: 9% for H20, 0% for H100, and 2% for S75 (Table 2). Nano-2 was then prepared, and the values for cost, viscosity, pH, size, and PDI were similar to those predicted (Table S2, supplementary material), validating the mathematical model.

#### 3.1.2. Characteristics of Prepared Nano-1 and Nano-2

Table 5 shows the physicochemical characteristics of Nano-1 and optimized Nano-2.

It can be observed in Table 5 that Nano-2 presented a droplet size almost three times larger than Nano-1. Nano-1, in turn, had a similar average size (*t*-test, *P* > 0.05) to that of NC (prepared only with mineral oil and synthetic surfactants).

Nano-1 and Nano-2 showed an apparent viscosity with the same order of magnitude and a pseudoplastic rheological behavior, with a slight reduction in viscosity as a function of the shear rate increasing and restoration of the initial viscosity with a decrease in shear rate (Supplementary Materials, Figure S2).

There was no significant change in the organoleptic characteristics (color and odor), size, PDI, viscosity, or pH after the preliminary stability test (freeze/unfreeze and centrifugation tests) (Supplementary Materials, Table S3).

**Figure 1:**
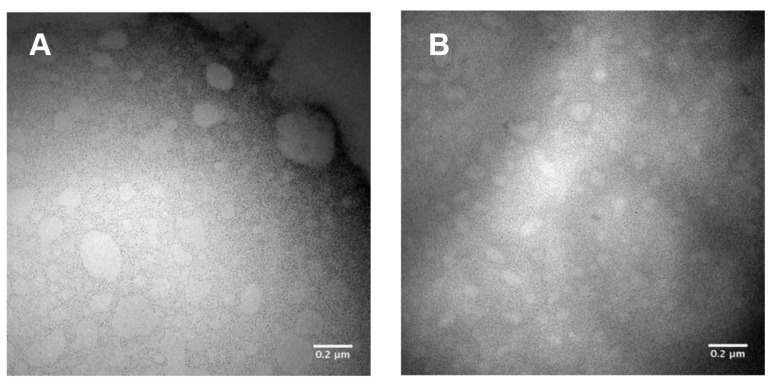
Representative images obtained by transmission electron microscopy of samples diluted 100 times in distilled water. Nano-1 (**A**) and Nano-2 (**B**). One hundred thousand times increase.

Figure 1 shows TEM representative images of nanoemulsions’ morphology.

The images show droplets with a well-defined outline, with an average diameter of 700 nm, more significant than that observed when the samples were analyzed by DLS, suggesting coalescence of the droplets during slide preparation.

### 3.2. In Vitro Studies in Cell Culture

**Figure 2:**
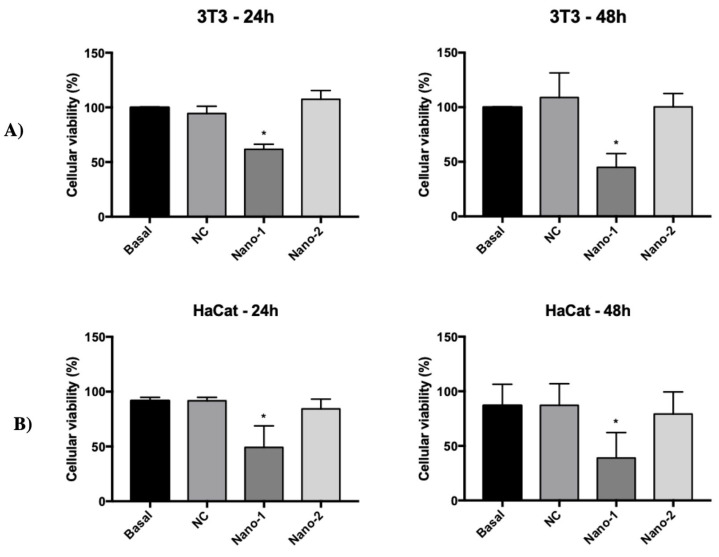
Viability of NIH-3T3 fibroblasts (**A**) and HaCat keratinocytes (**B**) after 24 h and 48 h incubation with nanoemulsions at 10^12^ particles/mL. Results were statistically analyzed using ANOVA, followed by Tukey’s test. (*) indicates statistically significant difference with *P* < 0.05 (n = 3 determinations).

Figure 2 shows the viability of fibroblasts (3T3) and keratinocytes (HaCat) after 24 and 48 h in contact with nanoemulsions at a concentration of 10^12^ particles/mL. Nano-2 and NC did not interfere with the cell viability of any cells under the conditions and times studied. Nano-1 also did not show cytotoxicity when incubated at 10^11^ nanoparticles/mL concentrations. In the range of 10^12^, however, Nano-1 decreased cell viability by 38 ± 4% and 51 ± 17% for fibroblasts and keratinocytes, respectively, after 24 h and by 55 ± 10% and 61 ± 20% after 48 h. None of the nanoemulsions altered the viability of the cells when they were treated for up to 15 min, regardless of the concentration of nanoparticles/mL.

**Figure 3:**
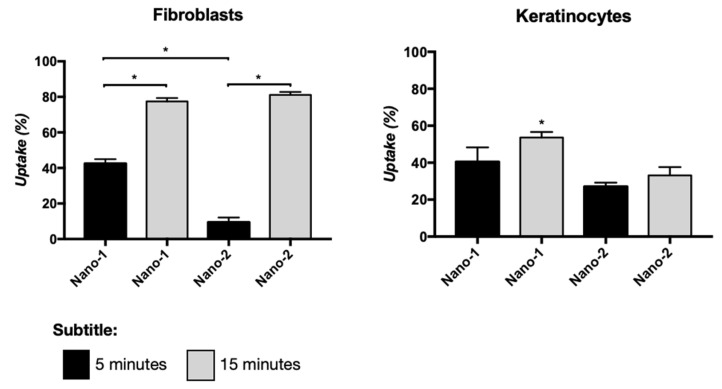
Cellular uptake of Nano-1 and Nano-2, at a concentration of 10^11^ particles/mL, after 5 and 15 min of contact with a monolayer of fibroblasts and keratinocytes. Results were statistically analyzed using ANOVA, followed by Tukey’s test. (*) indicates statistically significant difference with *P* < 0.05 (n = 3 determinations).

Figure 3 represents the internalization of Nano-1 and Nano-2 by fibroblasts and keratinocytes after 5 and 15 min of incubation. The uptake of Nano-1 by fibroblasts was faster than that of Nano-2, but after 15 min of incubation, both formulations showed a similar percentage of uptake at approximately 80%. In keratinocytes, the uptake percentage increased with incubation time only for Nano-1, resulting in 54 ± 2% uptake after 15 min. The uptake of Nano-2 was about 1.5 times lower than that of Nano-1 after the same incubation time.

**Figure 4:**
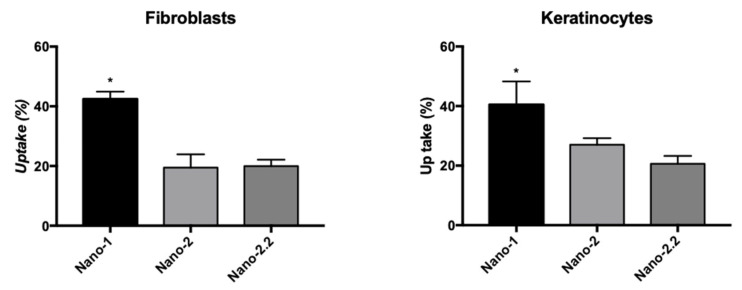
Cellular uptake of Nano-1 and Nano-2 compared to Nano-2.2, with nanoparticle size similar to that of Nano-1, after 5 min of contact with a monolayer of fibroblasts and keratinocytes. Results were statistically analyzed by ANOVA analysis of variance, followed by Tukey’s test. (*) indicates statistically significant difference with *P* < 0.05 (n = 3 determinations).

The differences observed in the uptake speed may be due to the distinct characteristics of Nano-1 and Nano-2, i.e., the composition of their emulsifying systems and the diameter of their droplets. To identify which of these characteristics most contributed to the result, Nano-2 was subjected to three additional cycles in the high-pressure homogenizer. This treatment led to obtaining a formulation with particles of approximately 180 nm, called Nano-2.2. Figure 4 shows the uptake of this nanoemulsion after 5 min of incubation with fibroblasts and keratinocytes compared to Nano-1 and Nano-2.

Decreasing the diameter of the Nano-2 droplets did not increase their uptake, suggesting that the difference in uptake percentage between Nano-1 and Nano-2 is related to the components of the emulsifying system. It also did not affect the distribution of the lipophilic marker BODIPY in the cells. Representative images of this distribution in keratinocytes and fibroblasts are shown in the supplementary material (Figure S3).

**Figure 5:**
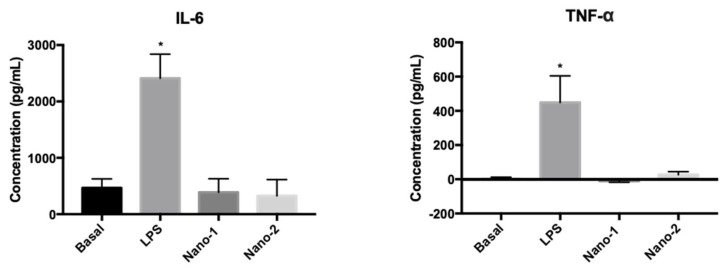
IL-6 and TNF-α production induced by treating human macrophages with LPS (positive control) and nanoemulsions. Results were statistically analyzed by ANOVA, followed by Tukey’s test. (*) indicates statistically significant difference with *P* < 0.05 (n = 3 determinations).

Figure 5 shows the IL-6 and TNF-α release by human macrophages extracted and isolated from three individuals and treated with nanoemulsions.

The treatment of macrophages with nanoemulsions at about 3 × 10^11^ particles/mL did not significantly change (ANOVA, *P* ≥ 0.05) the basal levels of the pro-inflammatory mediators IL-6 and TNF-α. On the other hand, treatment with LPS, as expected, significantly increased the production of these mediators. These results suggested that the studied formulations do not induce an inflammatory process.

### 3.3. In Vitro Penetration Studies Using Human Skin

**Figure 6:**
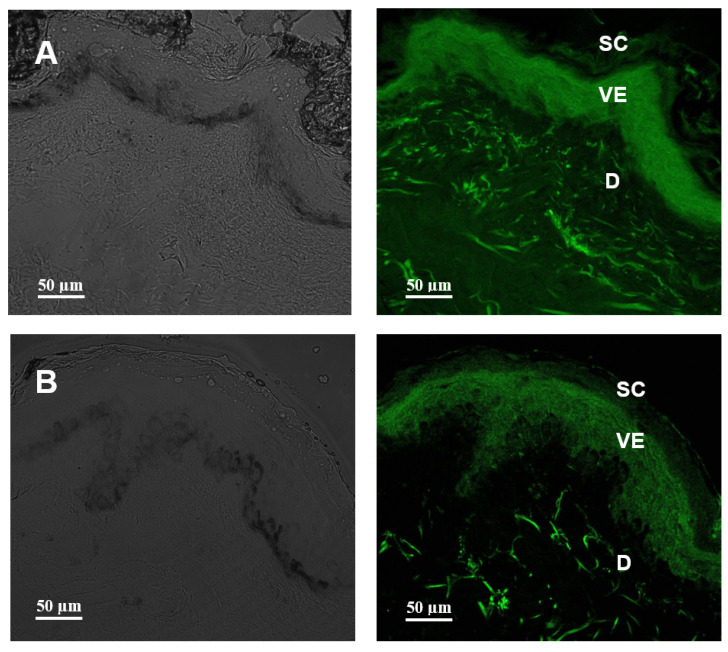
Confocal microscopy of human skin cross-sections treated for 1 h with Nano-1 (**A**) and Nano-2 (**B**). The green coloration is BODIPY marker’s distribution indicative in the different skin strata: stratum corneum (SC), viable epidermis (VE), and dermis (D). The left panel represents the bright field.

Figure 6 shows representative confocal microscopy images of human skin treated for 1 h with Nano-1 and Nano-2 labeled with the lipophilic fluorescent dye BODIPY. Nano-1 was distributed homogeneously throughout all skin layers (Figure 6A). Nano-2 penetration was restricted to the epidermis (Figure 6B).

### 3.4. Ex Vivo Studies in Human Organotypic Skin Explant Wound Model (hOSEC)

**Figure 7:**
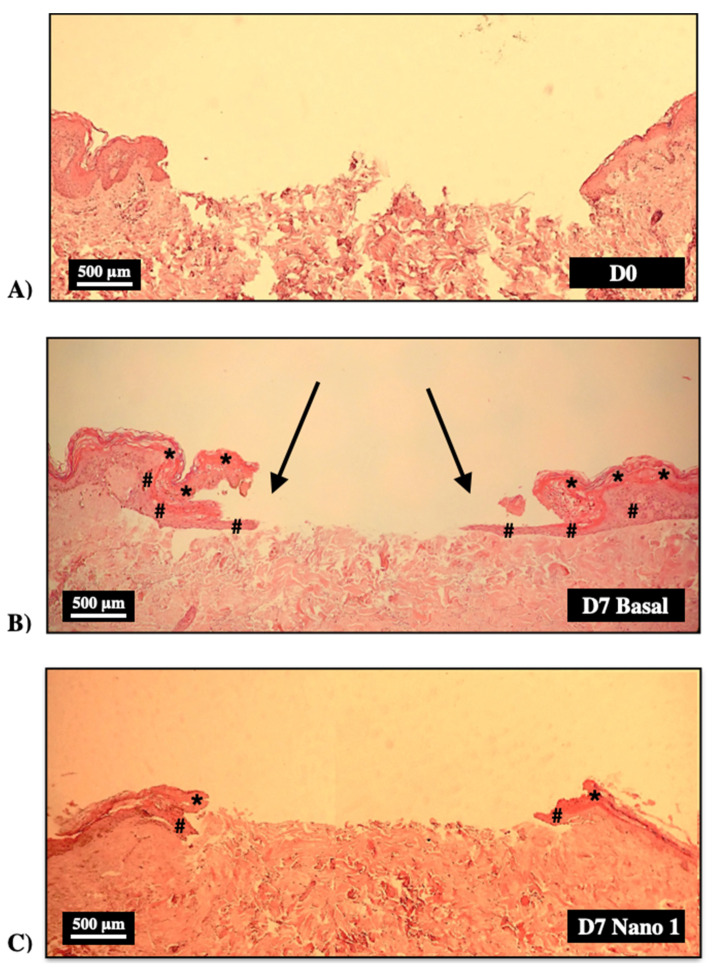
Histological sections of human skin fragments immediately (D0) after ulcer induction (**A**) and after 7 days (D7) kept in DMEM culture medium (**B**) and treated with daily application of Nano-1 without dilution (6.5 × 10^13^ particles/mL) (**C**). Hematoxylin-eosin staining. 100× magnification. Arrows show the lining of the ulcer by a new epithelium. * stratum corneum, # new epithelium.

Figure 7 shows histological sections of wounds made on human skin explants shortly after wound induction (D0) and after 7 days maintained in culture medium (Basal Group) and treated daily with Nano-1 without dilution (6.5 × 10^13^ particles/mL).

Keratinocyte migration and projection from the edges towards the interior of the wound (arrows in Figure 7B), giving rise to the known “tongue of keratinocytes”, can be observed in the explants not treated with the nanoemulsion (Baseline Group). However, the treatment with undiluted Nano-1 prevented this projection (Figure 7C).

**Figure 8:**
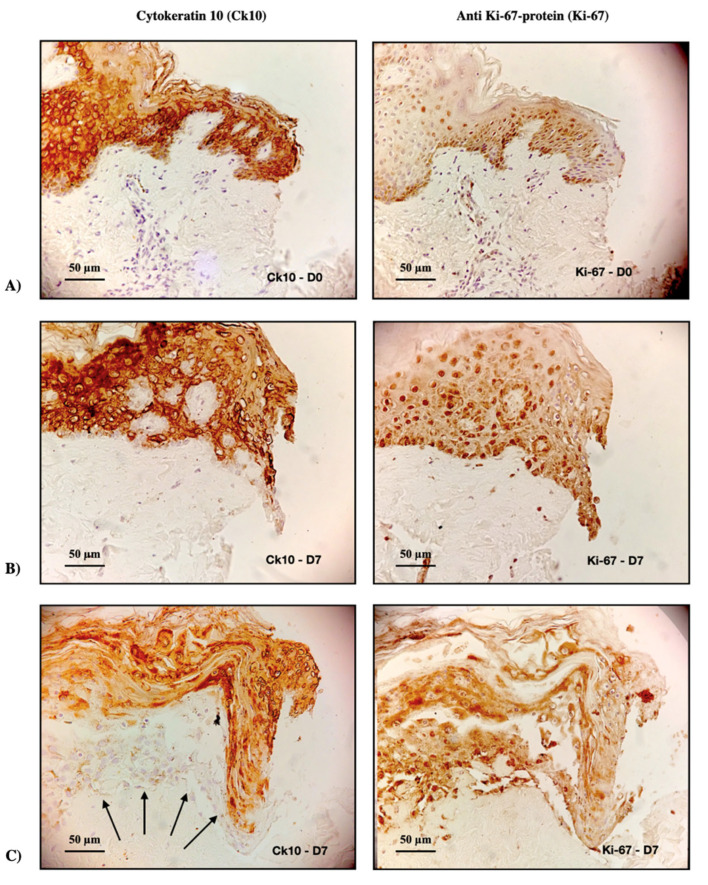
Immunohistochemical evaluation of the left ulcers’ edge in human skin fragments immediately after inducing the ulcer (**A**) and after 7 days (D7) treated daily with Nano-1 (**B**,**C**). The images on the left panel represent cytokeratin Ck10 stains (Ck10), and those on the right panel represent anti Ki-67-protein stains (Ki-67). Arrows indicate cells in the early stages of differentiation; 200× magnification.

To assess whether treatment with Nano-1 resulted in cell death, sections were stained with cytokeratin 10 (Ck10) and anti-Ki-67 protein (Ki-67), specific markers for differentiating cells and proliferating cells, respectively. Figure 8 illustrates these markers’ presence at the wound edge at baseline (D0) and after 7 days (D7) of daily treatment of skin fragments with undiluted Nano-1.

Cells from undiluted Nano-1 treated fragments were stained with Ck10 and Ki-67 (Figure 8), suggesting that the cells were viable. The difficulty of keratinocytes proliferating towards the ulcer’s center when they were treated with undiluted Nano-1 (Figure 7C) is correlated, therefore, with a physical blocking caused by the high concentration of nanoemulsion particles inside the induced wound.

**Figure 9:**
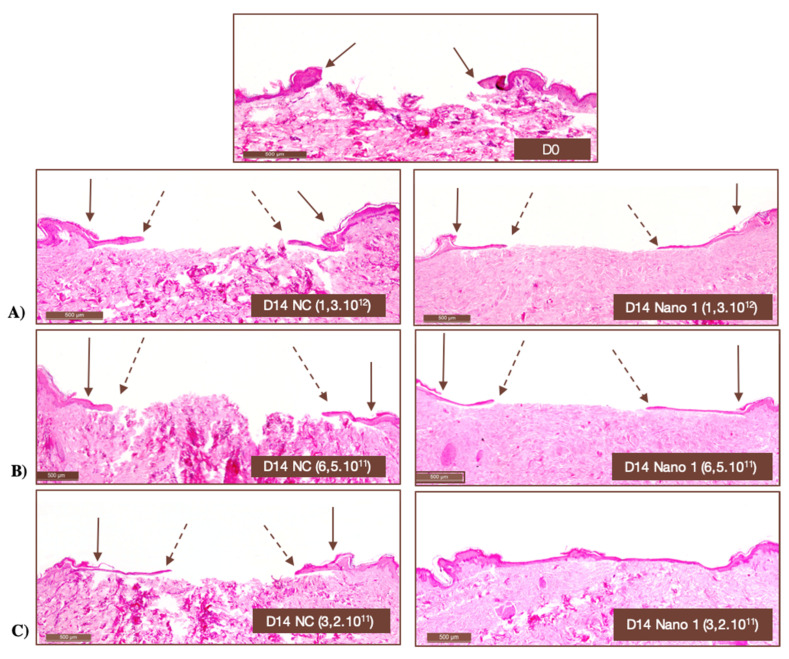
Histological images of human skin fragments immediately after ulcer induction (D0) and after 14 days of daily application of different numbers of NC and Nano-1 particles. (**A**) 1.3 × 10^12^ particles/mL, (**B**) 6.5 × 10^11^ particles/mL, and (**C**) 3.2 × 10^11^ particles/mL. Hematoxylin-eosin staining. 100× magnification. Solid arrows indicate the onset of ulceration, while dashed arrows indicate the lining of new epithelium.

Table 6 shows the percentage of wound closure as a function of the applied Nano-1 nanoparticle concentration and treatment time. In Figure 9, representative histological images of the skin fragments can be seen right after ulcer induction and after 14 days of treatment with different concentrations of Nano-1 and NC particles.

It can be seen in Table 6 that the ulcers treated with 3.2 × 10^11^ particles/mL of Nano-1 showed higher closure percentages than those treated with higher concentrations of nanoemulsion. Therefore, this nanoparticle density was chosen for the treatment of wounds with the Nano-2.

**Figure 10:**
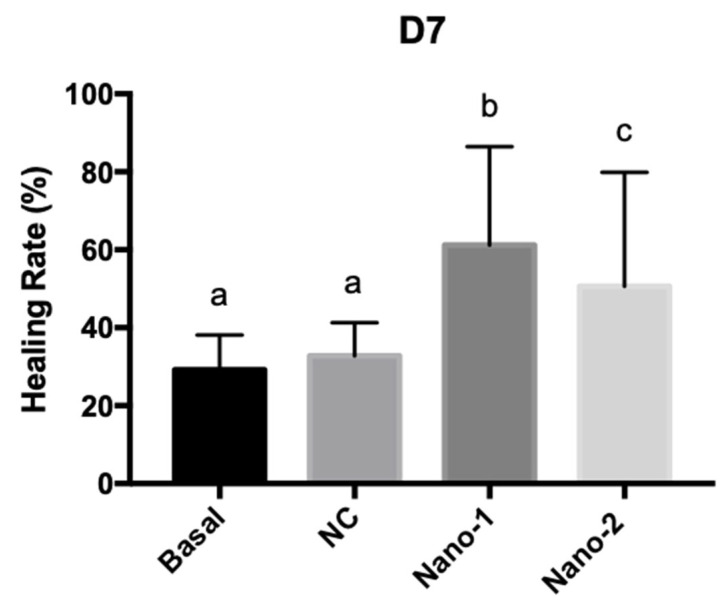
Graphic representation of the healing rates for human skin fragments treated daily for 7 days with culture medium (Basal), and 3.2 × 10^11^ particles/mL of mineral oil control nanoemulsion (NC), Nano-1, and Nano-2. Results were statistically analyzed by ANOVA, followed by Tukey’s test. Different letters indicate statistically significant differences with *P* < 0.05 (n = 3 determinations).

Figure 10 shows the percentage of wound healing after 7 days of daily treatment with the nanoemulsions administered at the same concentration of particles. Images of skin fragments taken before and after ulcer induction and 7 days after treatment with the formulations can be seen in the supplementary material (Figure S4).

Ulcers treated with nanoemulsions based on vegetable oils (Nano-1 and Nano-2) showed a significantly higher healing rate than those treated with mineral-oil-based nanoemulsions (NC) and those only kept in the culture medium. Additionally, the healing rate of wounds treated with Nano-1 was significantly higher than those treated with Nano-2.

## 4. Discussion

Despite all the therapeutic advances, the treatment of skin lesions still represents a significant challenge. High-cost products of scientifically unproven efficacy corroborate the alarming numbers of economic and personal losses inherent in wound-healing epidemiology [25]. Many available products are ineffective because the multifactorial aspect of the healing process needs to be considered in their development once the wound healing, regardless of etiology, constitutes a complex biological process, with simultaneous interaction of cellular, biochemical, and immunological events [26]. Thus, a product of healing wound action should stimulate, concomitantly, the recovery of the skin constituents, the control of bacterial and fungal infections, the modulation of tissue inflammation, and the interruption of the generation of free radicals. The latter may result from endogenous inflammatory processes [27] or the external environment, such as ionizing radiation in radiodermatitis.

In this context, the logical step for developing a wound-healing product should consider compounds with known antioxidant, anti-inflammatory, and antimicrobial action and with the potential to reestablish the original skin constituents. Such activities were considered in the choice of vegetable oils that formed the nanoemulsions developed in this work and their respective emulsifying systems. Furthermore, this study aimed to evidence the impact caused on the physicochemical and biological characteristics of nanoemulsions with different emulsifying systems composed of phospholipids and added low concentrations of a synthetic stabilizer (Nano-1, polysorbate 60).

The sunflower [28,29] and rosehip [30] oil concentrations were selected based on studies showing their biological activity in the concentrations used. Alpha-bisabolol was added to the formulation in adequate amounts to exert its antioxidant action [31].

To investigate the replacement of the synthetic surfactant of Nano-1 by another emulsifier source of phospholipids of natural origin, the experimental design with variations in the concentrations of H100 and S75 and the addition of H20, with at least 20% phosphatidylcholine, was designed to obtain Nano-2. Because it does not represent a raw material as pure as H100 and S75, H20 has a considerably lower cost. Thus, H100, S75, and H20 represented the independent variables of the experimental design elaborated to obtain Nano-2 (Table 2).

During the development of Nano-2, formulations with droplets of different sizes were found according to the composition of the emulsifying system of each manipulated formulation (Table 3). All of them, however, presented a diameter of less than 500 nm, most with narrow distribution, with PDI lower than 0.3, and can be considered nanoparticle systems suitable for topical administration [32]. This nanometric size range gives the dispersions kinetic stability, extending their shelf life, facilitating spreadability, and increasing the contact surface with the application site [33,34].

Viscosity and cost were the only variables significantly influenced by the emulsifying systems evaluated (Table 4). Thus, they were considered to define the most appropriate mathematical model to predict the characteristics of the final Nano-2 with the desired properties: stable, low cost, and low viscosity. More fluid formulations can be administered as a spray to treat difficult-to-reach and susceptible lesions, such as those affecting mucous membranes or burns. The ease of application and painless characteristics are decisive for patient compliance and treatment success. In addition, the increase in the viscosity of the formulations indicates the formation of other structures in the medium due to the combinations that can occur between the stabilizing agents. For example, the formation of micelles, liposomes, and even liquid-crystalline structures may occur when phospholipid mixtures are dispersed in an aqueous medium in high concentrations [14]. It was decided to keep the concentration of the stabilizers lower, working with smaller viscosity ranges, to prevent the formation of very complex delivery systems.

It can be observed in the mathematical equations defined from the experimental design (Table 4) that only the independent variables H20 and H100 influenced the viscosity of the formulations, increasing as a function of their concentration. Because it represents a mixture of phospholipids, H20 may favor the formation of phospholipids’ multilayers on the nanodroplets’ surface and alters the individual organization of the other emulsifiers at the oil–water interface, influencing the packaging and the rheology of the formulation [14,35].

Regarding the cost, it can be observed in the mathematical model equation (Table 4) that the three emulsifiers, H20, H100, and S75, influenced the final price of the nanoemulsion, but H100, due to the high purity in phosphatidylcholine (98%) extracted from the sunflower, increased the most the final cost of the formulation.

With the mathematical model and equations obtained from the experimental design, it was possible to perform numerical optimization and define the percentages of each emulsifier necessary to obtain the desired nanoemulsion. Given the range of desired values for viscosity (61 to 253 cP) and cost minimization, the optimal concentrations of each independent variable were obtained (Table 2) and used to prepare the Nano-2.

Nano-1 and Nano-2 were stable (Table S3, supplementary material), with viscosity in the same order of magnitude (Table 5). The size of the droplets varied depending on the mixture of emulsifiers. Nano-1, which contained the synthetic surfactant in addition to phospholipids, presented droplet size almost three times smaller than Nano-2 (Table 5), composed only of emulsifiers of natural origin. The largest droplet size of Nano-2 can explain the lower droplet concentration in each milliliter of the formulation, as seen in Table 5.

The largest size of Nano-2 may be related to the H20′s mixture of phospholipids, glycolipids, and triglycerides that compose the lecithin from the sunflower [36]. These components could be organized differently in the dispersion, affecting the nanoemulsion properties and changing its texture, appearance, or stability [37].

Although differences in the size and the organization of phospholipids at the oil–water interface may be possible, Nano-1 and Nano-2 were stable at room temperature and in preliminary thermal and mechanical stress tests (Supplementary Material, Table S3). When submitted to the drying process for TEM analysis (Figure 1), however, an increase in the size of the droplets was observed when compared with DLS measurements. This increase can probably be due to the evaporation of the external aqueous phase of the nanoemulsions, which can result in the rearrangement of the emulsifiers at the interface and the droplet coalescence as observed in the TEM images. Still, the morphology of the particles could be determined by TEM.

Furthermore, the nanoemulsions were formulated with more than 70% of water and with high hydrophilic–lipophilic balance (HLB) emulsifiers, with HLB ranging between 15 (Polysorbate 60) and 8 (sunflower lecithin [38], and Lipoid S75 [39]), conducive to forming oil-in-water emulsions. Despite these common characteristics, differences in the composition of stabilizers and droplet size of Nano-1 and Nano-2 could have impacted their interaction with fibroblasts and keratinocytes, cytotoxicity, and induction of inflammatory processes in the skin. Indeed, for the same density of nanoparticles, Nano-1 was more cytotoxic than Nano-2 for skin cells (Figure 2). This difference may be related to particle size and synthetic surfactant in Nano-1. However, the NC nanoemulsion obtained with synthetic surfactants and particle size similar to that of Nano-1, was not toxic to these cells under the conditions studied. Polysorbate 60, present in Nano-1, was more cytotoxic than polysorbate 80 [40], present in NC, suggesting that polysorbate 60 was partly responsible for the Nano-1 cytotoxicity. Nano-1′s smaller particle size could have facilitated the cellular uptake of the oils that made up the nanoemulsion’s internal phase and contributed to the more significant toxicity of this formulation. In fact, Nano-1 was more rapidly internalized by fibroblasts and keratinocytes than Nano-2 (Figure 3). After 15 min, nanoemulsions uptake by fibroblasts was similar, but keratinocytes internalized approximately 20% less Nano-2.

It has been demonstrated that different properties of nanoparticles, such as size, shape, material, and surface coating, influenced their cellular uptake [41]. To understand whether the different results obtained in the uptake assays were due to the composition of the emulsifying systems of Nano-1 and Nano-2 or to their different diameters, Nano-2 was subjected to long pressure cycles in the homogenizer until its droplet size reached that of Nano-1. The uptake of this nanoemulsion, denominated Nano-2.2, was not statistically different from that suffered by Nano-2, a nanoemulsion of identical composition but of distinct size (Figure 4). This result suggested that the chemical constitution of emulsifying systems and their arrangement on droplets’ surfaces was more relevant for the internalization of the nanoemulsions than their size.

Therefore, the Nano-1 synthetic surfactant was more available in the dispersing medium and had some permeabilization effect on the cell membranes, reflecting greater internalization of droplets (Figure 3) and toxicity (Figure 2). It should be noted, however, that these experiments were carried out in cell monoculture, which is much more sensitive than three-dimensional cell cultures or skin tissue.

The safety of the formulations was also evaluated for the induction of pro-inflammatory activity (Figure 5). Pro-inflammatory cytokines, such as IL-6 and TNF-α, can induce an imbalance in the reactive oxygen species production in the skin, delaying the wound-healing process [42]. It can be observed in Figure 5 that the nanoemulsions did not alter the levels of IL-6 and TNF-α from the macrophages, evidencing the nanoemulsions’ safety.

To evaluate the versatility of nanoemulsions, studies of skin penetration in vitro in healthy human skin and explants of human skin with induced ulcers were performed.

The studies on intact, healthy skin were conducted to evaluate the ability of nanoemulsions to favor the cutaneous penetration of sunflower and rosehip oils, potentiating their anti-inflammatory and antioxidant action in the skin and enabling their use in dermatitis treatment, for example. Figure 6 shows that Nano-1 allowed deeper penetration of the lipophilic dye incorporated into its oily phase compared to Nano-2. The smaller particle size of Nano-1 and its greater ease of internalization by keratinocytes (Figure 3 and Figure 4) could have contributed to this greater penetration.

On the other hand, Nano-2 maintained high concentrations of the lipophilic marker in the viable epidermis. This targeting is interesting in treating different skin diseases. The Nano-1’s penetration up to the dermis layer could allow the delivery of substances to the systemic circulation. Sometimes, this is not ideal for dermatological treatments due to undesired systemic effects and adverse events.

The potential of the formulations for treating cutaneous wounds was investigated in an induced-wound model in human skin explants. The influence of the density of nanoparticles added to the induced ulcer was initially evaluated to validate the model. Figure 9 clearly shows that the high density of oily nanodroplets (internal phase of the nanoemulsions) impaired wound closure in the ex vivo model. This impediment, however, was not related to the toxicity of the formulations since the expression of differentiating (Ck10) and proliferating cells (Ki-67) could be observed at the lesions’ edges (Figure 8), even when the nanoemulsion was applied without previous dilution.

This finding indicated that the nanoparticles inside the ulcer constitute a physical barrier that impaired the proliferation and migration of keratinocytes from the edges to the bed of the wound, preventing the progression of the “tongue of keratinocytes.” The “tongue of keratinocytes” is a line of proliferating keratinocytes on the dermis that originates in the viable epidermis adjacent to the wound. This new epithelium supports the development of the skin’s stratum corneum. The “snakehead” observed at the wound’s edge indicated the beginning of the proliferative phase of healing. Although the “snakehead” was present in samples treated with high concentrations of nanoparticles, the keratinocyte tongue was prevented from progressing, and the advance of epithelial cells to the center of the lesion was not observed. Thus, these results evidenced the fragility of the neoformed tissue and the need to work with a lower density of nanoparticles to investigate skin re-epithelialization in the hOSEC wound model.

Particle density in the range of 10^11^ particles/mL proved adequate for the progression of the keratinocyte tongue (Table 6 and Figure 9). The concentration of 3.2 × 10^11^ particles/mL allowed a faster wound closure and was standardized for subsequent experiments. However, it does not mean that nanoemulsions must be diluted for in vivo applications. These findings only suggest that controlling particle concentration of nanoparticulate delivery systems is essential for not impairing cell proliferation in hOSEC model.

To understand the influence of nanoemulsions based on vegetable oils and the emulsifying system on the wound-healing rate, ulcers were treated with Nano-1, Nano-2, and NC at the same nanoparticle concentration. The treatment with nanoemulsions of sunflower and rosehip oils significantly increased the healing rate compared to the control (untreated ulcer) (Figure 10). However, the ulcers treated with the mineral-oil-based nanoemulsion (NC) showed no significant difference from the control. Compared to the NC, the anti-inflammatory, antioxidant, and antimicrobial properties of the vegetable oils [2,43,44,45] of Nano-1 and Nano-2 may explain the enhanced wound healing results. Moreover, the lipids that constitute these oils can contribute to the re-establishment of the stratum corneum and the increase in the permeability of the cell’s membrane, facilitating the entry of growth factors and thus stimulating cell proliferation and migration and neoangiogenesis [9], also explaining the best healing rate compared to NC.

A difference was also observed between nanoemulsions based on vegetable oils. Despite having the same sunflower and rosehip oil concentrations, Nano-1 slightly increased the healing rate compared to Nano-2. The emulsifying system of the Nano-1 could also have influenced the wound healing process by stabilizing the droplets in smaller sizes and enhancing the oils’ delivery to the target cells.

Altogether, both nanoemulsions were promising drug delivery systems for lipophilic molecules, with Nano-1 targeting to deeper layers of the skin and Nano-2 directing delivery to the epidermis layer. The ex vivo studies with the hOSEC wound model suggested that Nano-1 possessed more potential in treating wounds. Nano-2 could be applied to treat dermatological disorders, such as atopic dermatitis and psoriasis. 

## 5. Conclusions

In summary, vegetable oils were successfully emulsified in stable nanoemulsions, with adequate viscosity for spray administration, using phospholipid emulsification extracted from sunflower and soybean. Adding a small percentage of a synthetic surfactant to the phospholipid-emulsifying system resulted in nanoemulsions with smaller diameters, greater cellular uptake and skin permeation, and more significant healing potential. Nanoemulsions composed only of phospholipids of natural origin as emulsifiers and stabilizers could target the penetration of vegetable oils to the viable epidermis and presented a lower cytotoxic potential than the nanoemulsions containing the synthetic surfactant. Furthermore, the importance of standardizing the number of nanoparticles for the ex vivo hOSEC wound model was shown for the first time in this study to ensure reliable responses in wound healing tests. In this ex vivo wound model, the vegetable-oil-based nanoemulsions of smaller size and stabilized with a small concentration of a synthetic emulsifier besides the phospholipids emulsifiers were more efficient in wound healing. The appropriate combination of phospholipids of natural origin can allow the obtaining of vegetal-based nanoemulsions based with specific and versatile characteristics and potential applications for the treatment of skin disorders.

## Figures and Tables

**Table 1:** Box–Behnken experimental design for Nano-2 development using a combination of phospholipids extracted from sunflower (H100 and H20) and soybean (S75).

Formulations		A	B	C
F1		−1	−1	0
F2		+1	−1	0
F3		−1	+1	0
F4		+1	+1	0
F5		−1	0	−1
F6		+1	0	−1
F7		−1	0	+1
F8		+1	0	+1
F9		0	−1	−1
F10		0	+1	−1
F11		0	−1	+1
F12		0	+1	+1
F13		0	0	0
F14		0	0	0
F15		0	0	0
**Coded** **Variables**	**Decoded** **Variables**	**Levels**		
		**−1**	**0**	**+1**
A	H20	8	10	12
B	H100	0	0.5	1
C	S75	2	2.5	3

**Table 2:** Composition of Nano-1 and optimized Nano-2.

Components	Concentration (% *w*/*w*)	
	Nano-1	Nano-2
Sunflower oil	15	15
Rosehip oil	3	3
Alpha-bisabolol	0.3	0.3
Polysorbate 60	3	-
Lipoid S75	1	2
Lipoid H100	0.5	-
Lipoid H20	-	9
Glycerin	5	5
Water (enough quantity to)	100	100

**Table 3:** Physicochemical characteristics and cost of Nano-2 prototypes as a function of independent variables (percentages of stabilizers H20, H100, and S75).

Nanoemulsions Characteristics—Nano-2 Prototypes						
Formulations	Size (nm)	PDI	Viscosity (cP)	Emulsification Rate (%)	pH	Cost (USD) *
F1	392	0.430	61	37	5.8	206.83
F2	388	0.340	477	100	4.3	248.62
F3	387	0.272	116	66	5.7	260.41
F4	488	0.283	5819	100	5.9	302.20
F5	384	0.337	109	81	5.5	208.97
F6	353	0.257	1932	100	6.1	250.76
F7	339	0.232	72	100	5.6	258.27
F8	385	0.269	1596	100	5.4	300.06
F9	391	0.278	75	100	5.6	203.08
F10	370	0.306	426	100	5.4	256.66
F11	304	0.197	355	100	5.7	252.37
F12	367	0.270	253	100	5.7	305.95
F13	342	0.232	166	100	6.0	254.52
F14	358	0.250	378	100	5.7	254.52
F15	403	0.310	385	100	5.6	254.52

* Costs of 1 Kg of phospholipid mixture. The costs represent the conversion into USD of the CIF (Cost, Insurance, and Freight) price for Brazil, provided by Lipid Ingredients & Technologies, Lipoid GmbH’s official representative and distributor in Latin America.

**Table 4:** Statistical analysis of the estimated effect of the independent variables on the viscosity and cost of formulations obtained using phospholipids extracted from sunflower (H100 and H20) and soybean (S75) as emulsifiers for sunflower and rosehip oils.

Statistical Analysis		Responses (Dependent Variables) (Y)			
		Viscosity (Y1)		Cost (Y2)	
		F Value	*P*-Value	F Value	*P*-Value
Prediction Model		19.44	0.0001	36314.19	0.0001
Coded independent variables *	A	50.90	<0.0001	43604.97	<0.0001
	B	7.36	0.0202	44356.63	<0.0001
	C	0.653	0.8030	20980.95	<0.0001
Equations		Log_10_(Y1) = −1.1 + 0.3A + 0.5B + 0.15C		Y2 = 0.2 + 10.45A + 53.58B + 49.30C	
R^2^ adjusted		0.7981		0.9999	
R^2^ predicted		0.6801		0.9998	

* The coded independent variables A, B, and C refer to H20, H100, and S75 used in the nanoemulsion formulation, respectively.

**Table 5:** Physicochemical characteristics of Nano-1 and optimized Nano-2.

Parameter	Nano-1	Nano-2
Size (nm)	130 ± 3	370 ± 15
PDI	0.197 ± 0.025	0.228 ± 0.040
Number of particles/mL	6.5 × 10^13^ ± 1.7 × 10^12^	3.8 × 10^13^ ± 1.42 × 10^12^
Apparent viscosity (cP)	20 ± 3	79 ± 6
pH	5.4 ± 0.2	5.7 ± 0.2
Cost (USD) *	81.33	192.85

The size and number of NC particles were 180 ± 2 nm and 5.9 × 10^13^ ± 0.2 × 10^13^ particles/mL. Results are expressed as mean ± standard deviation. * Costs of 1 Kg of emulsifier mixture.

**Table 6:** Wound closure percentage as a function of the number of Nano-1 particles applied and treatment time. n = 3 determinations.

Number of Particles/mL of Nano-1	% Wound Closure about D0 *	
	Day-7	Day-14
1.3 × 10^12^	43 ± 9	28 ± 1
6.5 × 10^11^	48 ± 2	75 ± 25
3.2 × 10^11^	66 ± 26	76 ± 17

* D0: immediately after ulcer induction.

## Data Availability

The data presented in this study are available on request from the corresponding author.

## Supplementary Materials

The following supporting information can be downloaded at https://www.mdpi.com/article/10.3390/pharmaceutics15030999/s1. Figure S1: 3D graphs (A) and response surface graphs (B) for viscosity versus concentration in (%) of factors H20 and H100. Figure S2: Rheological behavior of preparations. In red, the viscosity values related to the increase in shear rate, and in blue, values obtained after shear reduction. Figure S3: Representative images obtained by confocal microscopy of the uptake assay of the Nano 1 (left panel) and Nano 2 (right panel) formulations after 15 min of contact with HaCat keratinocyte cells (A) and NIH-3T3 fibroblasts (B). λ_exc/em_ = 477/512 nm for visualization of bodipy in green, λ _exc/em_ = 405/413–472 nm for visualization of cell nucleus—DAPI in blue. Figure S4: Representative images of graphic in Figure 10 obtained by histological images of human skin fragments after 7 days (D7) in culture, without ulcer (A), and with ulcer receiving 5 µL of daily treatments: Basal (B), NC (C), Nano-1 (D), and Nano-2). Table S1: Significance results of the most suitable mathematical models found after ANOVA for each dependent variable (Software Design-Expert 11.1.0 © 2018 Stat-Ease). Table S2: Experimental data obtained after preparing the Nano-2 with the optimized composition and the statistical prediction given by the mathematical model. Table S3: Physical–chemical characterization of nanoemulsions 24 h after preparation and followed by preliminary stability test.
