# Supplementary material for: Nanoemulsions Based on Sunflower and Rosehip Oils: The Impact of Natural and Synthetic Stabilizers on Skin Penetration and an Ex Vivo Wound Healing Model
Source: Pharmaceutics. 2023 Mar 20;15(3):999. doi: 10.3390/pharmaceutics15030999 (PMC10053715; doi:10.3390/pharmaceutics15030999)
Supplement: Supplementary file 1 [file pharmaceutics-15-00999-s001.zip › pharmaceutics-2188286-supplementary.pdf]

pharmaceutics

MDPI

Supplementary Material

Nanoemulsions based on Sunflower and Rosehip Oils: The Impact of Natural and Synthetic Stabilizers on Skin Penetration and an Ex vivo Wound Healing Model

Cynthia Nara Pereira Oliveira [1], Marcel Nani Leite [2], Natália Aparecida de Paula [2], Yugo Araújo Martins [1], Sonia Aparecida Figueiredo [3], Marco Andrey Cipriani Frade [2] and Renata Fonseca Vianna Lopez [1,*]

[1] School of Pharmaceutical Sciences of Ribeirão Preto, University of São Paulo, Ribeirão Preto 14040-903, SP, Brazil; cynthianara@ufmg.br (C.N.P.O.); yugomartins@usp.br (Y.A.M.);
[2] Department of Internal Medicine, Faculty of Medicine of Ribeirão Preto, University of São Paulo, Ribeirão Preto 14049-900, SP, Brazil; marcelnani@gmail.com (M.N.L.); npbiomed@yahoo.com.br (N.A.d.P.); sonia.figueiredo@unifal-mg.edu.br (S.F.); mandrey@fmrp.usp.br (M.A.C.F.)
[3] Department of Food and Drug, School of Pharmaceutical Sciences, Federal University of Alfenas, Alfenas 37130-001, MG, Brazil; sonia.figueiredo@unifal-mg.edu.br (S.A.F.);
* Correspondence: rvianna@fcfrp.usp.br; Tel.: +55-16-3315-4202

Table S1: Significance results of the most suitable mathematical models found after ANOVA for each dependent variable (Software Design-Expert 11.1.0 © 2018 Stat-Ease)

Dependent Variables	Mathematical Model	Significance (P)*
Emulsification rate (%)	Quadratic	> 0.05
Size (nm)	Quadratic	> 0.05
PDI	Average	> 0.05
Viscosity (cP)	Linear (log)	< 0.05
pH	Interaction of two factors	> 0.05
Cost ($/Kg)	Linear	< 0.05

Table S2: Experimental data obtained after preparing the Nano-2 with the optimized composition and the statistical prediction given by the mathematical model

Responses	Mathematically predicted values	Experimentally obtained values*
Size (nm)	374 ± 26	369 ± 15
PdI	0.284 ± 0.06	0.23 ± 0.04
pH	5.6 ± 0.4	5.7 ± 0.2
Viscosity (cP)	80 ± 3	79 ± 6
Cost (U$)	192.85 ± 0.09	192.85

*Mean ± standard deviation from 3 independent experiments

Table S3: Physical-chemical characterization of nanoemulsions 24 h after preparation and followed by preliminary stability test

Characteristic	Nanoemulsion			
	Nano-1		Nano-2	
Collor	White		Light beige	
Viscosity (cP)	20 ± 3		79 ± 6	
Odor	Characteristic		Characteristic	
pH	Pre stability test	5.4 ± 0.2	Pre stability test	5.7 ± 0.2
	Post stability test	5.5 ± 0.3	Post stability test	5.8 ± 0.3
Size (nm)	Pre stability test	130 ± 3	Pre stability test	363 ± 15
	Post stability test	130 ± 5	Post stability test	332 ± 14
PDI	Pre stability test	0.197 ± 0.025	Pre stability test	0.228 ± 0.040
	Post stability test	0.210 ± 0.012	Post stability test	0.185 ± 0.056

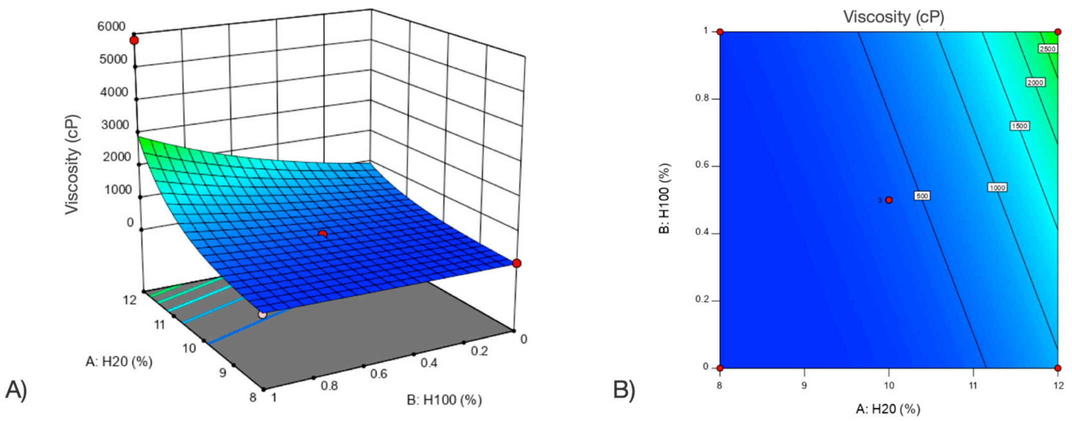

Figure S1: 3D graphs (A) and response surface graphs (B) for viscosity versus concentration in (%) of factors H20 and H100.

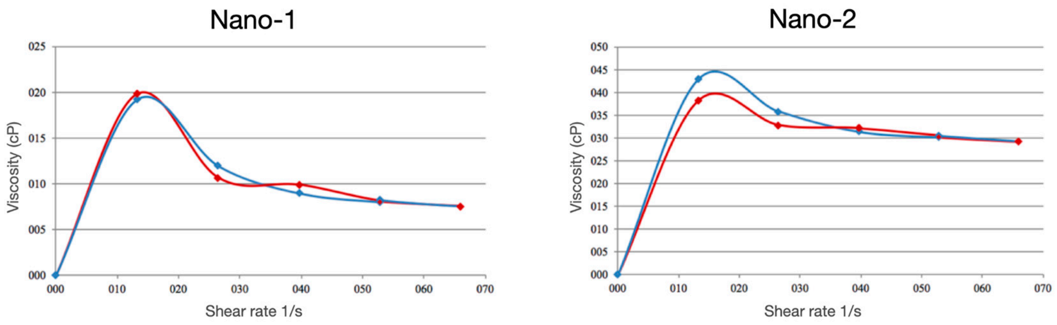

Figure S2: Rheological behavior of preparations. In red, the viscosity values related to the increase in shear rate and in blue, values obtained after shear reduction.

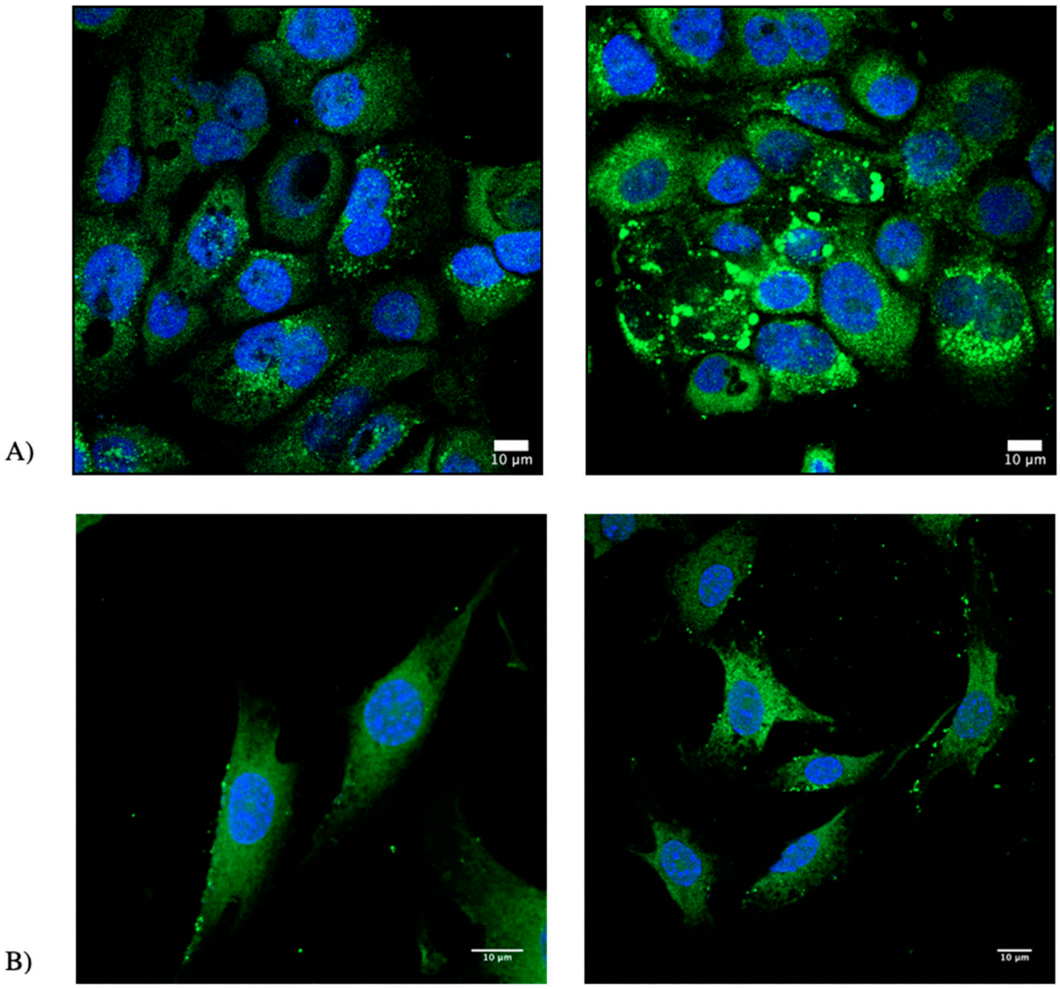

Figure S3: Representative images obtained by confocal microscopy of the uptake assay of the Nano 1 (left panel) and Nano 2 (right panel) formulations after 15 minutes of contact with HaCat keratinocyte cells (A) and NIH-3T3 fibroblasts (B). $\lambda_{exc/em}$ = 477/512 nm for visualization of bodipy in green, $\lambda_{exc/em}$ = 405/413-472 nm for visualization of cell nucleus - DAPI in blue.

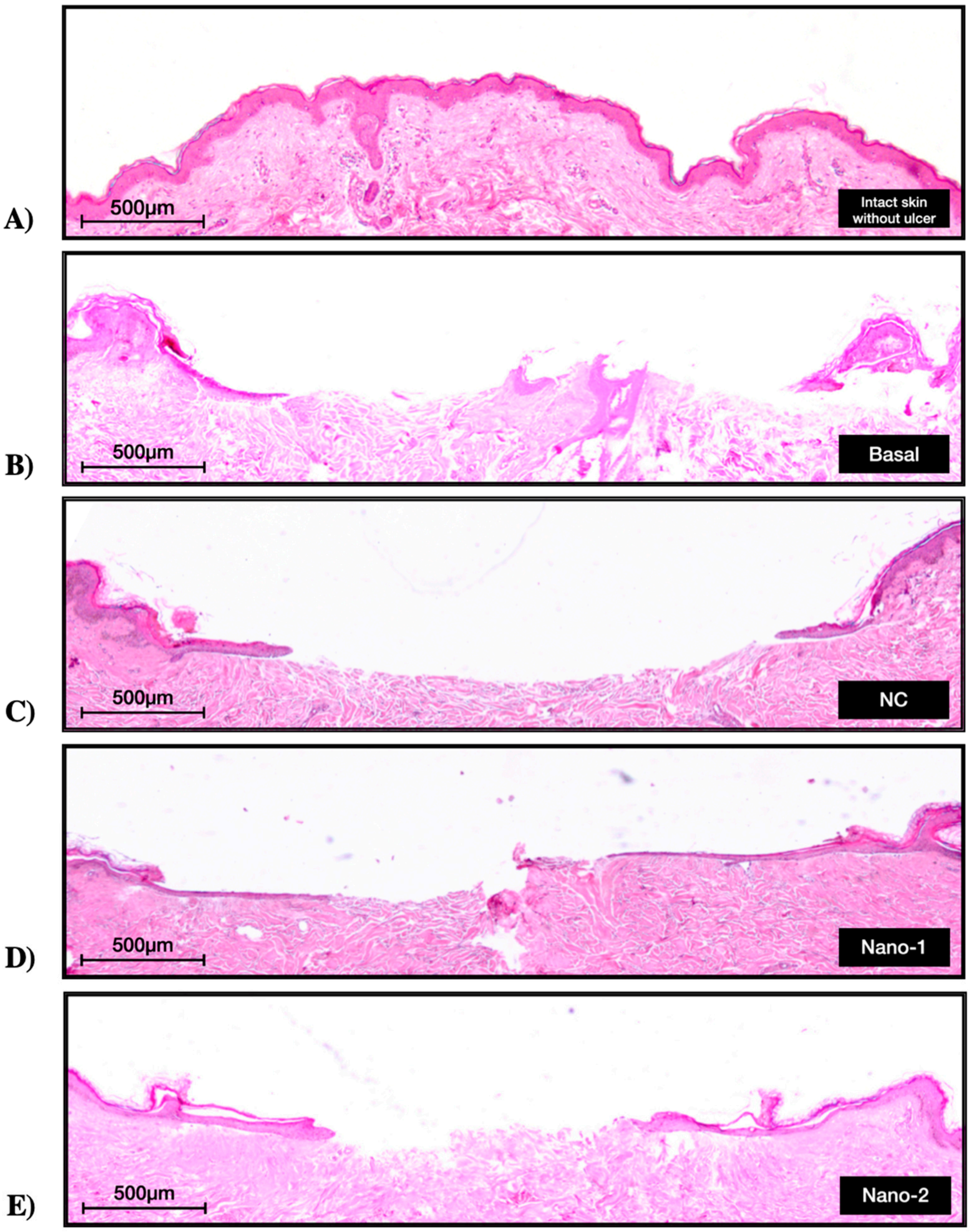

Figure S4: Representative images of graphic in Figure 10 obtained by histological images of human skin fragments after 7 days (D7) in culture, without ulcer (A), and with ulcer receiving 5 µl of daily treatments: Basal (B), NC (C), Nano-1 (D), and Nano-2 (E).
